# Moving closer towards a comprehensive view of tumor biology and microarchitecture using spatial transcriptomics

**DOI:** 10.1038/s41467-023-42960-6

**Published:** 2023-11-02

**Authors:** Young Min Park, De-Chen Lin

**Affiliations:** 1 Center for Craniofacial Molecular Biology, Herman Ostrow School of Dentistry, and Norris Comprehensive Cancer Center, University of Southern California, Los Angeles, CA USA; 2 Department of Otorhinolaryngology, Yonsei University College of Medicine, Seoul, South Korea

**Keywords:** Cancer genomics, Cancer genomics, Cancer microenvironment

## Abstract

Spatial transcriptomic profiling of cancer has enabled spatial delineation of malignant transcriptional heterogeneity, intercellular communication, and organization of microanatomical structures within the tumor microenvironment. This technical breakthrough paves the way for the development of precision diagnostic methods and targeted therapies.

Spatial transcriptomics has emerged as a powerful tool in cancer research, allowing for the visualization of gene expression patterns within the spatial context of tumor tissues. Methodologically, there is a wide range of such technologies available today^1^, which can be broadly categorized into sequencing-based (e.g., Visium, GeoMx, Stereo-seq) and imaging-based methods (e.g., MERFISH, Xenium, CosMx). Sequencing-based spatial methods offer advantages such as high scalability, plexity, and short scanning time of large areas. However, they have relatively lower RNA capture efficiency and often do not achieve single-cell resolution. In comparison, imaging-based technologies provide subcellular resolution with high RNA capture efficiency. Nevertheless, technical limitations of imaging-based methods include modest-to-limited scalability, optical crowding, and long imaging time. Therefore, selecting the most suitable method depends on specific research goals, spatial resolution requirements, sensitivity and detection limit, as well as properties of the tissue or samples^1^.

Intratumoral heterogeneity and functional plasticity of malignant cells is a cancer hallmark, enabling tumor evasion from immune surveillance/attack, adaptation to metabolic constraints, as well as treatment resistance^2^. For example, the transition between epithelial-mesenchymal states is crucial for tumor metastasis and drug resistance^2^. Notably, spatial transcriptomic profiling has shown that cellular plasticity and intratumoral heterogeneity are strongly dependent on spatial context^3–5^.

To spatially delineate intratumoral transcriptional heterogeneity, the computational pipeline typically begins with the deconvolution of cellular composition and identification of malignant spots using supervised machine learning based on curated training data. Subsequently, regional transcriptional programs (or “gene modules”) are identified. A clustering approach next takes gene module scores as input values to identify consensus transcriptional programs that can be horizontally integrated across patient tumor samples. A correlative analysis then uses shared transcriptional programs as anchors to perform spatially-weighted correlations to explore the colocalization of intratumoral transcriptional programs. Spatial transcriptomic investigations have successfully captured intratumoral transcriptional programs established by single-cell RNA sequencing (scRNA-seq). For example, in gliomas, scRNA-seq-defined four cellular states:^6^ mesenchymal-like, neural progenitor-like, astrocyte-like, and oligodendrocyte precursor-like, were all identified by a spatial study using the Visium platform^5^. Importantly, distinct cellular states appear to be spatially segregated. Indeed, perhaps unsurprisingly, EMT (epithelial-mesenchymal transition)-like cancer cells tended to inhabit along the leading front versus tumor core, demonstrated by Visium-based investigations^3,7^. Glioma cells expressing a reactive-hypoxia program occupied the necrotic edge, which might reflect ongoing metabolic stress^5^. In breast cancer, different subclone territories exhibited distinguishing transcriptional and histological characteristics, which can be observed even in ductal carcinoma in situ, a precancerous lesion^4^. This spatially organized pattern of intratumoral transcriptional heterogeneity and cellular state is somewhat surprising. Indeed, considering the stochastic accumulation of genomic aberrations during clonal evolution, one might expect tumor regions to be a mosaic of various cellular states. Thus, the micro-regional segregated pattern indicates the spatial constraint as a powerful selection pressure in shaping the intratumoral transcriptional heterogeneity. However, technical limitations (e.g., detection sensitivity, resolution limit, and cluster assignment) should be kept in mind since they may partially contribute to the lack of detection of the mosaic pattern.

This remarkable spatial segregation of cancer cell states begs an important question: what are the underlying molecular mechanisms? By developing a base-specific in situ sequencing technology (BaSISS)^4^ to spatially co-map gene mutations and transcription at the single-cell level in breast cancer samples, Lomakin et al. demonstrate that genomic diversification is at least partially responsible. Indeed, the authors spatially resolved the locations of many genetically-defined subclones, and charted the relationships between genomic and transcriptional patterns. Perhaps the most striking finding is that virtually every sample displayed spatial segregation and organization of multiple genetic subclones (albeit only a total of 10 tissue samples were profiled), exhibiting clone-specific gene expression accompanied by distinct stromal surroundings^4^. These observations are echoed by a spatial transcriptomic map of human glioblastoma samples, wherein the researchers investigated the relationship between copy number aberrations and spatially distinct transcriptional programs. Upon reconstruction of clonal architecture using patient-specific hierarchical clustering of copy number aberrations, Ravi et al.^5^ noted that cells expressing a reactive-hypoxia program, which were spatially segregated, harbored distinguishable and more complex chromosomal alterations, compared with other spatial spots. Consistently, orthogonal experimentation revealed that chronic hypoxia culture of patient-derived glioblastoma lines induced a significant accumulation of copy number aberrations than normoxia, establishing a causal relationship between hypoxia and genomic instability. Additionally, the hypoxia condition led to promoter DNA hypermethylation of the *MGMT* gene^5^. These results underscore the complex interplay between local metabolic stress, intratumoral transcriptional adaptation, and genomic diversification, in a highly spatial-dependent manner.

Beyond neoplastic cells, scRNA-seq profiling has comprehensively and systematically cataloged many stromal and immune cell types in the tumor microenvironment. However, these works only provide circumstantial information on how cells communicate and interact with each other. Spatial transcriptomic analyses have begun to reveal the characteristics of various forms of cell-cell communications over different proximity length scales, such as direct interactions, short-range, and even far-range interactions.

The crosstalk involving immune cells is perhaps hitherto the most extensively studied. A pattern repeatedly seen is the immune-suppressive microenvironment frequently characterized by interactions between tumor cells, different T-cell subsets, and suppressive macrophages^3,8^. For example, in skin squamous cell carcinoma, a tumor-specific keratinocyte population displayed both autocrine and paracrine interactions with macrophages and myeloid-derived suppressor cells, among other cell types^3^. In an imaging mass cytometry analysis of breast cancer^8^, enriched interactions between Treg cells, exhaustive T cells, and macrophages were found in an immune-suppressive niche.

In addition to immune cells, fibroblasts often show dynamic spatial distribution and interaction patterns, likely owing to their highly versatile and plastic functional states. Indeed, different fibroblastic cell subsets appear to have different preferences in their spatial localization. In breast cancer characterized by the Visium method, myofibroblast-like fibroblasts resided along tumor peripherals while certain immunoregulatory fibroblasts were observed to disperse across stroma- and immune-enriched niche^9^. Various ligands from immunoregulatory fibroblasts were identified to mediate the communication with T cell receptors nearby. Unsurprisingly, interacting partners of fibroblasts are reciprocally versatile. In colorectal cancer, for example, Visium-generated data showed that FAP^+^ fibroblasts resided in close proximity to SPP1^+^ macrophages, and their interaction led to a desmoplastic reaction and inhibition of immune cell infiltration^10^.

It should be noted, however, that not all cells in close proximity are having biological interactions. In fact, data from 3D high-resolution optical analyses of morphological and physical features suggests that only a minority of spatially co-localizing cells may have direct crosstalk^11^. Indeed, about 20% of immune cells in close contact with melanoma cells showed likely biological interactions featured by cell surface molecular polarization. The same study also identified that some macrophages could extend inhibitory synapses with CD8^+^ T cells which were at least over one cell diameter away (>10μm), highlighting that non-adjoining cells can establish functional contacts with one another^11^. These emerging observations suggest that spatial proximity is neither sufficient nor necessary for biological cell-cell communications, underscoring the complex nature of cellular crosstalk in the cancer ecosystem. On the other hand, this research has also emphasized the necessity for the development of deep learning-based computational vision algorithms to fully automate the analysis of high-resolution, high-plex tissue images, which currently rely partially on manual inspection and interpretation.

At the millimeter scale, large multi-cellular organizations have been observed in the tumor ecosystem as spatially segregated compartments and microanatomical structures, sometimes involving three or more distinct cell types^1^. In general, tumors organize into a compartment dense with malignant cells, while a stromal compartment is often formed predominantly by immune cells, fibroblasts, endothelial cells, and extracellular matrix (ECM). The interface between these two compartments, also known as the leading edge, is characterized by distinct cellular composition, ECM makeup, and distribution^1^. Biologically, the tumor-stromal border appears to contribute to the regulation of anti-tumor immune response^11,12^. Indeed, in melanoma, a consolidated and spatially confined immune-suppressive microenvironment was identified along the tumor-stromal interface, featuring melanoma cells making simultaneous contact with both CD8^+^ T cells and Treg cells^11^. The tumor-stromal boundary of breast cancer was enriched with PD-L1^+^ myeloid cells and MHCII^+^ tumor cells, indicative of an immune-suppressive structure, as revealed by a multiplexed ion beam imaging method^12^. The leading edge is also implicated in the regulation of tumor cell invasion and infiltration. For example, the aforementioned skin cancer subpopulation, characterized by expressing gene programs associated with EMT and cellular migration/invasion, was localized specifically at the leading edge^3^. A similar spatial pattern was also seen in other cancer types, such as head and neck cancer^7^.

Tertiary lymphoid structures (TLSs) are spatially organized aggregates of immune cells, including a B cell-rich center zone surrounded by T cells, dendritic cells, and macrophages. TLSs are mainly observed in tissues exposed to chronic inflammation but have also been identified in various tumor types. TLSs within the tumor microenvironment appear to be a result of ongoing immune responses against the tumor, serving as sites for immune cell activation, proliferation, and interaction^13^. Recent spatial methodologies have revealed greater granularity of the TLS structure and highlighted their biological significance in anti-tumor immune responses. For example, a Visium-based profiling of renal tumors showed that the entire process of B cell maturation could be completed within TLSs^14^. By focusing on the architecture of TLSs and their spatial vicinity, this study demonstrated that mature plasma cells, disseminated along the fibroblastic track in the tumor microenvironment, produced IgG antibodies which caused cancer cell apoptosis. Moreover, therapeutic responses and patient survival were correlated with IgG-bound tumor cells in renal cancer patients treated with immune checkpoint inhibitors^14^. Importantly, recognizing that the Visium method (capturing 3′ RNA sequence) is not best suited for sequencing the BCR repertoire, the authors validated some of the key findings using complementary 5′ bulk RNA-seq.

The field of spatial cancer transcriptomics is experiencing rapid growth and evolution, uncovering unprecedented molecular and spatial details of tumor biology. Moving forward, integrating spatial transcriptomics data with other omics technologies, including genomics, proteomics, and metabolomics, will enable the simultaneous analysis of different layers of spatial regulation, providing a truly comprehensive view of the molecular landscape of tumors. Advancements in companion computational methods, including deep learning algorithms, will facilitate the identification of complex spatial patterns, molecular interactions, and predictive models for tumor behavior and treatment response. Another exciting front of spatial cancer biology is the development of spatial genetic perturbation using animal models^15,16^, which can reveal the direct functionality of spatially regulated gene expression or mutations. Indeed, early works such as Perturb-map, an approach combining spatial transcriptomics (using the Visium platform) with in vivo CRISPR screens, have already linked certain gene knockouts to spatial immune responses in the tumor microenvironment^16^. Overall, the future of spatial cancer research holds immense potential to advance our understanding of tumor biology, guide personalized medicine approaches, and drive the development of innovative therapeutic strategies for cancer treatment.
